# Concatenation of Observed Grasp Phases with Observer’s Distal Movements: A Behavioural and TMS Study

**DOI:** 10.1371/journal.pone.0081197

**Published:** 2013-11-20

**Authors:** Elisa De Stefani, Alessandro Innocenti, Doriana De Marco, Maurizio Gentilucci

**Affiliations:** 1 Department of Neuroscience, University of Parma, Parma, Italy; 2 RTM (Rete Tecnologica Multidisciplinare), IIT (Istituto Italiano di Tecnologia) and Università di Parma, Parma, Italy; University G. d'Annunzio, Italy

## Abstract

The present study aimed at determining how actions executed by two conspecifics can be coordinated with each other, or more specifically, how the observation of different phases of a reaching-grasping action is temporary related to the execution of a movement of the observer. Participants observed postures of initial finger opening, maximal finger aperture, and final finger closing of grasp after observation of an initial hand posture. Then, they opened or closed their right thumb and index finger (experiments 1, 2 and 3). Response times decreased, whereas acceleration and velocity of actual finger movements increased when observing the two late phases of grasp. In addition, the results ruled out the possibility that this effect was due to salience of the visual stimulus when the hand was close to the target and confirmed an effect of even hand postures in addition to hand apparent motion due to the succession of initial hand posture and grasp phase. In experiments 4 and 5, the observation of grasp phases modulated even foot movements and pronunciation of syllables. Finally, in experiment 6, transcranial magnetic stimulation applied to primary motor cortex 300 ms post-stimulus induced an increase in hand motor evoked potentials of opponens pollicis muscle when observing the two late phases of grasp. These data suggest that the observation of grasp phases induced simulation which was stronger during observation of finger closing. This produced shorter response times, greater acceleration and velocity of the successive movement. In general, our data suggest best concatenation between two movements (one observed and the other executed) when the observed (and simulated) movement was to be accomplished. The mechanism joining the observation of a conspecific’s action with our own movement may be precursor of social functions. It may be at the basis for interactions between conspecifics, and related to communication between individuals.

## Introduction

When executing in succession different actions which are functionally interrelated, the execution of the previous action influences the next one [1–4]. The same occurred even when the previous action was observed. Indeed, evidence [5–10] suggests that the observation of actions affected actual actions executed by the observer. Specifically, interactions of biological distal effectors (hand, mouth and foot) rather than non-biological stimuli with differently sized objects influenced the kinematics of successive observer’s interactions with the same objects using the same or different effectors [7–9]. This suggested that the action was automatically simulated by the observer and mnemonic traces of the simulation influenced the actual action.

Moreover, the simultaneous production of two actions with different distal effectors is subjected to reciprocal influence [11] and the same occurred even when one of the two actions was observed and the other was executed [12–14]. The authors of the above cited studies found that when observing the reaching-grasping of differently sized objects, the simultaneous pronunciation of syllables was influenced by the different grasp. Voice parameters of syllables increased when observing the grasp of a large rather than small object. They interpreted these results as due to automatic simulation of the observed action during which the motor commands to the hand were also sent to the speaking mouth. This may explain the variation in voice spectra.

However, concerning the temporal relations between observation and execution of two movements a problem remains unsolved, that is whether (i) a synchronism relates simulation of observed distal movement with actual own distal movement or (ii) a mechanism couples in succession simulation of observed commands to distal effectors with actual commands to own distal effectors, on the basis of memorized motor patterns. Having assumed that the appropriateness of an observed grasp phase to trigger a successive movement of the observer could be shown by a decrease in Response Times (RTs) and/or an increase in kinematic parameters of the actual movement (e.g. acceleration and velocity), we reasoned that a synchronism between simulation of grasp and actual distal movement should have produced decreasing RTs and increasing speed when simultaneously observing and executing corresponding movement phases (e.g. observing the posture of initial finger aperture and executing finger opening). Conversely, if the commands were sent in succession, according to the idea that actions are liable to functional concatenation to each other because controlled by the same system [4,15,16], decreasing RT and increasing speed of actual movement should be found when starting to move during observation of the final phases of the observed action, that is when the action was likely to be accomplished. 

In the present study we aimed to determine whether the observation of various grasp phases of the reach-grasp action modulated RTs and kinematics of a successive observer’s movement. In other words, we verified whether the observation induced a motor simulation which was responsible for the beginning of the successive movement. We analyzed the effects of observed grasp phases (finger opening, maximal finger aperture, finger closing) on execution of finger (opening and closing) movements (experiments 1, 2 and 3), foot movements (lowering and lifting, experiment 4) and speech (syllable pronunciation, experiment 5). Finally in experiment 6, we controlled whether observation of grasp phases induced motor simulation; by using transcranial magnetic stimulation (TMS) technique, we verified the presence and degree of hand primary motor area (M1) activation during observation of grasp phases. In the case of concatenation of actual movement with observed movement, we expected activation of opponens pollicis muscle (OP), more evident (higher motor evoked potentials, MEPs) when observing the final phase of finger closing. This phase might be responsible for signalling the beginning of the successive movement. Conversely, Gangitano et al. [17] found increase in activation of First Dorsal Interosseus (FDI) when observing finger opening during reaching-grasping which was greater when finger aperture was maximal.

## Experiment 1

We studied the effects of observing phases of grasping a fruit on finger movements of opening and closing executed by the observer. 

### Methods

#### Participants

Fourteen volunteers (10 females, 20-30 years) participated in experiment 1, after providing written informed consent. All participants were right-handed, as ascertained by the Edinburgh Handedness Inventory [18]. All participants were naïve as to the purpose of the study. The Ethics Committee of the Medical Faculty at the University of Parma approved the experiment, which was carried out according to the declaration of Helsinki. 

#### Apparatus, stimuli, and procedure

**Figure 1:**
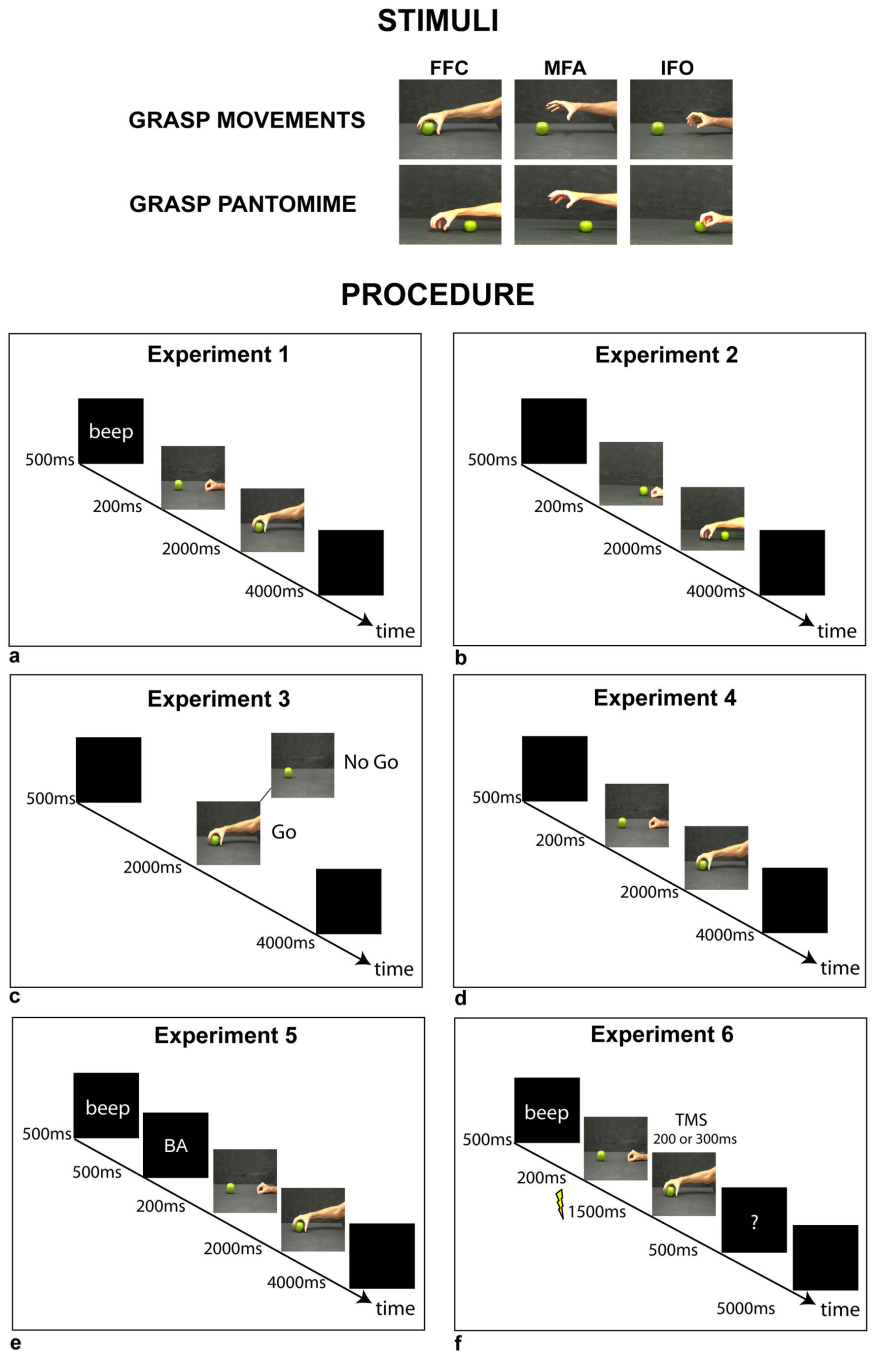
Stimuli and procedure of experiments 1-6. Upper panels show stimuli presented in experiments 1,3,4,5 (first row, grasp movements) and in experiment 2 (second row, grasp pantomime). Lower panels show the procedures of experiments 1-6.

The participant seated in front of a table with their right hand resting upon a support which allowed comfortable finger movements. Each trial began presenting a sound (BEEP; duration 500 ms). Then, on a PC monitor (19 inches) placed on the table, 60 cm distant from the participant’s chest, the participant was presented with one picture of grasp posture corresponding to one of the following grasp phases: initial finger opening (IFO), maximal finger aperture (MFA), final finger closing (FFC) on an object (either an apple or a peach using a Whole Hand Grasp, Figure 1). Each picture whose duration of presentation was 2000 ms was preceded by another picture in which the hand was presented in initial pinch position (200 ms duration). The rapid succession of presentation of the two pictures could induce the illusion of a grasp movement moving from the initial pinch position to the finger posture in that phase (apparent motion). At the beginning of the trial (before picture presentation), participants were required by the experimenter to open or close their right thumb and index finger. After stimulus presentation, as soon as they judged that the hand was still, they had to move. The required movement was opening or closing the fingers, according to the initial request of closing or opening. Explicitly, when initially the participants were required to open the fingers, they had to close them in response to picture presentation. Conversely, they had to open the fingers when initially required to close them. After picture presentation a blank panel was presented for 4000 ms before the successive trial. Stimuli presentation and beginning of movement recording using the SMART system (see below) were controlled by a software developed using MATLAB version 6.5 (R13). 60 trials were randomly run, 10 for each condition (two participant’s finger movements, opening versus closing, and three pictures of grasp phases, IFO versus MFA versus FFC.

#### Movement recording and data analysis

The kinematics of the finger opening and closing was recorded using the 3D-optoelectronic SMART system (BTS Bioengineering, Milano, Italy). This system consists of six video cameras detecting infrared reflecting markers (spheres of 5-mm diameter) at a sampling rate of 120 Hz. Spatial resolution of the system is 0.3 mm. The infrared reflective markers were attached to the nails of the participant’s right thumb and index finger. The data of the recorded movements were analysed using a software developed using MATLAB version 7.7 (R2008b). Recorded data were filtered using a Gaussian low pass smoothing filter (sigma value, 0.93). The time course of finger opening and closing was visually inspected: the beginning of the movement was considered to be the first frame in which the distance between the two markers placed on the right finger tips increased (finger opening) and decreased (finger closing) more than 0.3 mm (spatial resolution of the recording system) with respect to the previous frame. The end of the movement was the first frame in which the distance between the two right fingers increased/decreased less than 0.3 mm with respect to the previous frame. We measured for successive statistical analysis RT (Response Time), that is the time from the presentation of the picture to the beginning of finger opening or closing, and in addition peak acceleration and peak velocity of finger opening and closing. 

Repeated measures ANOVAs were carried out on RT, finger peak acceleration, and finger peak velocity. The within-subjects factors were finger movement (opening versus closing) and grasp phases (IFO versus MFA versus FFC), whereas the between subjects factor was condition (experiment 1 versus 2, see below). In all analyses post-hoc comparisons were performed using the Newman-Keuls procedure. The significance level was fixed at p=0.05.

## Experiment 2

We controlled whether the salience of the visual stimulus (hand close to the fruit) played a role in possible effects of grasp phases on actual observer’s movement.

### Methods

#### Participants

A new sample of fourteen volunteers (12 females, 20-40 years) participated in the experiment after providing written informed consent. All were right-handed, as ascertained by the Edinburgh Handedness Inventory [18]. All participants were naïve as to the purpose of the study. The Ethics Committee of the Medical Faculty at the University of Parma approved the experiment, which was carried out according to the declaration of Helsinki. 

#### Apparatus, stimuli and procedure

Apparatus and procedure were the same as in experiment 1. Stimuli were pictures of the same phases of the grasp as in experiment 1 (i.e. IFO, MFA, FFC) but, unlike experiment 1 the grasp was pantomimed. The fruit (either the apple or the peach) was placed close to the location of the finger initial posture instead of the location of the final finger closing (Figure 1). Presentation of the stimuli was as in experiment 1.

#### Movement recording and data analysis

Repeated measures ANOVAs were carried out on RT, finger peak acceleration, and finger peak velocity of experiments 1 and 2. The within-subjects factors were finger movement (opening versus closing) and grasp phases (IFO versus MFA versus FFC), whereas the between subjects factor was condition (experiment 1 versus 2). That is, we compared the effects of fruit in a congruent or incongruent position with the grasp. In all analyses post-hoc comparisons were performed using the Newman-Keuls procedure. The significance level was fixed at p=0.05. 

### Results and Discussion

**Figure 2:**
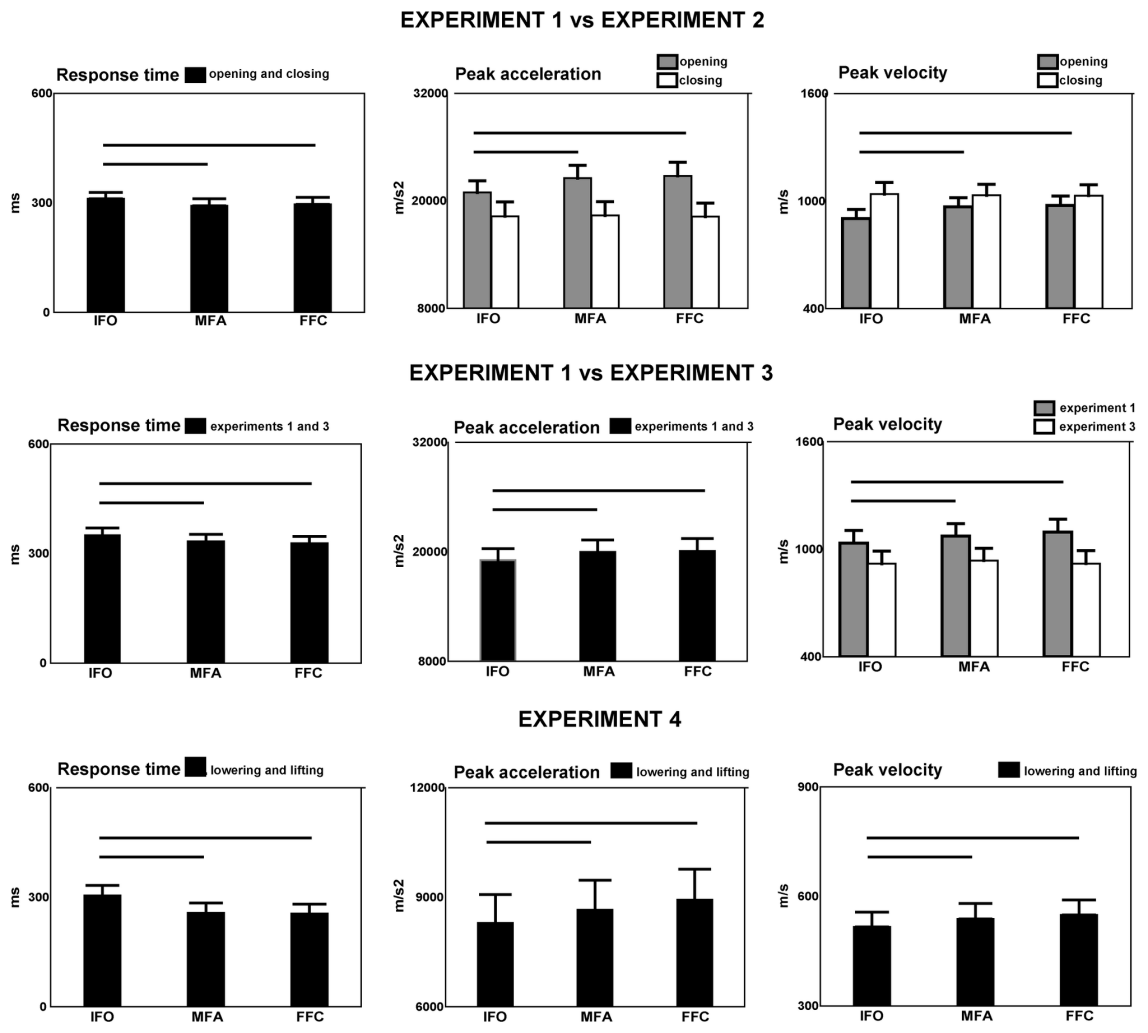
Mean RT and kinematic parameters (mean peak acceleration and mean peak velocity) of finger opening and closing (experiments 1, 2 and 3) and foot-tip lowering and foot-tip-lifting (experiment 4) executed after presentation of three phases of grasp. **IFO, initial finger opening, MFA, maximal finger aperture, FFC, final finger closing**. Horizontal lines show significance in ANOVA and vertical lines are SE.

The mean values of the parameters collected in experiments 1 and 2 are reported in Table 1. RT was significantly slower in IFO than in MFA and FFC phases (factor grasp phases: F(2, 52)=3.7 p=0.03, MFA versus IFO, p=0.04, FFC versus IFO, p=0.048, MFA versus FFC, p=0.62, Figure 2). Peak acceleration decreased in IFO as compared to MFA and FFC phases (factor grasp phases: F(2, 52)=4.1, p=0,021). This effect was found only during finger opening (interaction between finger movement and grasp phases, F(2,52)=6.3, p=0,004, finger opening, MFA versus IFO, p=0.0003, FFC versus IFO, p=0.0002, MFA versus FFC, p=0.53; finger closing, MFA versus IFO, p=0.82, FFC versus IFO, p=0.92, MFA versus FFC, p=0.94). The same occurred for peak velocity. It decreased in IFO as compared to MFA and FFC (factor grasp phases: F(2, 52)=3.6, p=0.03), but only during finger opening (interaction between finger movement and grasp phases, (F(2,52)=8.3, p=0.001, finger opening, MFA versus IFO, p=0.0002, FFC versus IFO, p=0.0002, MFA versus FFC, p=0.66; finger closing, MFA versus IFO, p=0.66, FFC versus IFO, p=0.84, MFA versus FFC, p=0.9, Figure 2). 

**Table 1:** Means and SE of kinematic and vocal parameters collected in experiments 1- 5.

		**APERTURE**						**CLOSURE**					
		IFO		MFA		FFC		IFO		MFA		FFC	
		*Mean*	*SE*	*Mean*	*SE*	*Mean*	*SE*	*Mean*	*SE*	*Mean*	*SE*	*Mean*	*SE*
**EXPERIMENT 1**	RT (ms)	336,1	28,85	305,32	29,32	302,53	28,29	274	24,9	252,65	26,04	241,84	26,8
	Peak Acceleration (mm/s^2^)	22618,9	1767,64	24226,3	2021,74	24967	2298,24	19281,6	1955,4	20032,7	2039,31	20428,2	2080,03
	Peak Velocity (mm/s)	996,23	64,33	1032,03	67,25	1061,64	74,81	1103,17	67,21	1114,45	70,2	1128,41	72,65
**EXPERIMENT 2**	RT (ms)	342,13	26,96	323,4	30,04	336,59	28,85	292,63	22,62	287,09	27,46	302,69	28,89
	Peak Acceleration (mm/s^2^)	19237,1	1951,3	20839,2	2012,43	20591,1	1986,5	17280,4	2516,91	16713,1	2321,87	16056,1	2154,93
	Peak Velocity (mm/s)	839,7	77,95	905,25	74,79	889,29	74,26	975,77	109,36	950,97	100,13	932,96	97,24
**EXPERIMENT 3**	RT (ms)	411,03	23,02	399	29,01	400,14	28,35	389,23	26,65	376,84	28,1	367,46	28,85
	Peak Acceleration (mm/s^2^)	19575,9	2174,42	20591,5	2259,7	20305,7	2295,88	14914,1	1592,74	15129,6	1623,69	14616,2	1634,16
	Peak Velocity (mm/s)	896,4	81,07	921,6	79,88	910,05	83,16	942,31	78,17	952,23	75,23	930,33	76,27
**EXPERIMENT 4**	RT (ms)	300,65	37,91	254,13	30,43	254,68	28,87	308,9	23,34	261,27	25,48	256,55	24,72
	Peak Acceleration (mm/s^2^)	8253,93	685,2	8517,92	735,65	8794,24	712,73	8345,13	913,32	8793,51	917,79	9077,77	1014,51
	Peak Velocity (mm/s)	556,88	42,63	572,74	41,25	592,5	43,19	475,66	41,95	504,35	45,11	506,1	42,63
**EXPERIMENT 5**		**BA**						**DA**					
		IFO		MFA		FFC		IFO		MFA		FFC	
	F1	*Mean*	*SE*	*Mean*	*SE*	*Mean*	*SE*	*Mean*	*SE*	*Mean*	*SE*	*Mean*	*SE*
		905,38	12,52	911,11	12,46	914,69	12,3	889,42	11,36	894,75	12,47	894,79	11,49

IFO = Initial Finger Opening MFA = Maximal Finger Aperture FFC = Final Finger Closing

 RT increased when opening compared to closing the fingers (324 ms versus 275 ms; factor finger movement: F(1,26) = 45.6, p=0.00001). Peak acceleration increased when opening (22080 mm/s^2^) than closing the fingers (18489 mm/s^2^, factor finger movement: F(1, 26)=13.54, p=0.001), whereas peak velocity decreased (949 versus 1034 mm/s; factor finger movement: F(1,26)= 5.7, p=0.02).

In both experiments 1 and 2 the various phases of the observed grasp movement differentially affected actual finger movement. The movement (RT, peak acceleration, and peak velocity) was quicker during observation of the FFC phase as compared to the IFO phase. Moreover, this effect was frequently observed even during observation of MFA phase. The results of experiment 2 ruled out the possibility that the closeness of the hand to the fruit during the final grasp phases could have made the visual stimulus more salient, attracted the observer’s attention and induced a quicker response to the stimulus. Indeed in experiment 2, in which the pictures of the pantomimes of IFO, MFA, and FFC were presented, the fruit was located close to the initial hand position (instead of the final one) and the participants observed the pantomime of the grasp. If the closeness of the hand to the fruit was responsible for quicker responses, we should have found different effects in experiment 1 as compared to experiment 2; that is quicker movements after presentation of MFA and FFC in experiment 1 and after the presentation of IFO in experiment 2. This was not found. 

It is possible that the observation activated the simulation of the action which initially interfered with (and/or in the final phases facilitated) the movement of another individual (i.e. the observer). In other words, when the observed action could be accomplished, the actual movement of the observer was facilitated. This effect (see below) was more evident when opening rather than closing the fingers probably because closing command contrasted with the contemporaneous simulated final posture of finger closure. However, it is unclear whether simulation was induced by the observation of an apparent motion (the two pictures were presented in rapid succession) or the presentation of a hand posture was sufficient. This issue was addressed in experiment 3, in which we presented the sole hand posture not preceded by any initial hand posture. In other words, we tested whether either the biological (apparent) movement participated in or the hand posture was sufficient for an effect on the successive movement. 

## Experiment 3

We aimed at controlling whether the observation of static hand posture was sufficient to produce an effect on finger opening/closing parameters.

### Methods

#### Participants

A different sample of fourteen volunteers (11 females, 21-38 years) participated in the experiment after providing written informed consent. All were right-handed, as ascertained by the Edinburgh Handedness Inventory [18]. The Ethics Committee of the Medical Faculty at the University of Parma approved the experiment, which was carried out according to the declaration of Helsinki.

#### Apparatus, stimuli and procedure

Apparatus and procedure were the same as in experiment 1. Stimuli were pictures of the same grasp phases as in experiment 1 (i.e. IFO, MFA, FFC). Unlike experiment 1, the picture of the hand in starting position was not presented. Other stimuli were the fruits (either apple or peach) presented alone. The participants performed a go-no-go task. Specifically, they executed the finger opening or closing movement when the hand posture was presented, whereas they were required to refrain to move in presence of the fruit alone. The remaining procedure was as in experiment 1.

#### Movement recording and data analysis

Repeated measures ANOVAs were carried out on RT, finger peak acceleration, and finger peak velocity of experiments 1 and 3. The within-subjects factors were finger movement (opening versus closing) and grasp phases (IFO versus MFA versus FFC), whereas the between subjects factor was condition (experiment 1 versus 3). That is, we compared the effects of static postures with effects of apparent motion of grasping hands. In all analyses post-hoc comparisons were performed using the Newman-Keuls procedure. The significance level was fixed at p=0.05.

### Results and Discussion

In Table 1 the mean values of the parameters collected in experiments 1 and 3 are reported. RT was slower in IFO condition than in MFA and FFC (factor grasp phases: F(2, 52)=5.8, p=0.005, MFA versus IFO, p=0.015, FFC versus IFO, p=0.01, MFA versus FFC, p=0.47, Figure 2). Peak acceleration of finger opening decreased in IFO as compared to MFA and FFC phases (factor grasp phases: F(2, 52)=5.2, p=0.008, MFA versus IFO, p=0.001, FFC versus IFO, p=0.014, MFA versus FFC, p=0.8, Figure 2). The same was found for peak velocity of finger opening which decreased in IFO as compared to MFA and FFC (factor grasp phases: F(2, 52)=4.0, p=0.025, MFA versus IFO, p=0.02, FFC versus IFO, p=0.04, MFA versus FFC, p=0.8), but only in experiment 1 (interaction between grasp phase and experiment, F(2,52)=3.1, p=0.05, experiment 1, MFA versus IFO, p=0.027, FFC versus IFO, p=0.002, MFA versus FFC, p=0.206, experiment 3, MFA versus IFO, p=0.56, FFC versus IFO, p=0.96, MFA versus FFC, p=0.33, Figure 2). 

RT increased when opening compared to closing the fingers (359 ms, 317 ms; factor finger movement: F(1,26) = 27.2, p=0.00002). Peak acceleration increased when opening the fingers (opening, 22048 mm/s^2^, closing, 17400 mm/s^2^, factor finger movement: F(1, 26)=21.2, p=0.00009), whereas peak velocity showed a trend to decrease (965 versus 1028 mm/s; factor finger movement: F(1,26)= 4.0, p=0.051). RT was shorter in experiment 1 than 3 (285 versus 391 ms; factor experiment: F(1,26), p=8.7, p=0.006,).

The observation of hand postures induced effects similar to those of the apparent motion even if some differences between the two experiments were found. First, in experiment 3, the effect of the grasp phases was observed on RT and peak acceleration, but not on peak velocity. Second, these effects were not selective for finger opening movement, unlike in the comparison of experiment 1 with 2. Consequently, we have no definitive confirm that the effect in experiment 1 was selective for finger aperture. In contrast, we are confident that it was selective in experiment 2, whereas it was not in experiment 3. 

## Experiment 4

We addressed the problem of whether it exists a more general effect of observed movement phase on executed movement of the observer, that is of an observed hand movement on an executed movement with another effector (e.g. foot). Specifically, we verified whether the observation of the hand grasp phases affected the observer’s foot tip movements of lowering and lifting.

### Methods

#### Participants

A different sample of fourteen volunteers (6 females, 21-40 years) participated in the experiment after providing written informed consent. All were right-handed, as ascertained by the Edinburgh Handedness Inventory [18]. The Ethics Committee of the Medical Faculty at the University of Parma approved the experiment, which was carried out according to the declaration of Helsinki.

#### Apparatus, stimuli and procedure

The participant seated in front of a table with his/her right foot resting upon a platform on the floor (20.5 cm high). It allowed comfortable foot movements and easier foot movement recording. Stimuli and their presentation were the same as in experiment 1. At the beginning of the trial (before picture presentation), the participants were required either dorso-flexing their right foot (“to lift the foot tip”) or placing their foot plant on the platform plane (“to lower the foot tip”). Then, a posture of hand grasp phase was presented after an initial pinch posture. As soon as the participants judged that the hand was still, they had either to lower (plantar-extending) or to lift (dorso-flexing) their foot tip. In total, 60 trials were randomly run, 10 for each condition (two participant’s foot movements, lowering versus lifting, and three pictures of grasp phases, IFO versus MFA versusFFC).

#### Movement recording and data analysis

The kinematics of the foot movements was recorded using the 3D-optoelectronic SMART system (BTS Bioengineering, Milano, Italy). The infrared reflective markers were spheres of 10-mm diameter. The markers were attached to the foot-tip, to the heel, and to the shin of the participant. The time course of the distance between the markers placed on the shin and foot tip was visually inspected: the beginning of the movement was considered to be the first frame in which the distance between the markers placed on the shin and foot tip increased (foot-tip lowering) or decreased (foot tip lifting) more than 0.3 mm (spatial resolution of the recording system) with respect to the previous frame. The end of the movement was the first frame in which the distance between the two markers increased/decreased less than 0.3 mm with respect to the previous frame. We measured for successive statistical analysis RT, that is the time from the presentation of the picture to the beginning of foot-tip lowering or foot-tip lifting, and in addition peak acceleration and peak velocity of foot lowering and lifting.

Repeated measures ANOVAs were carried out on RT, foot peak acceleration, and foot peak velocity. The within-subjects factors were foot movement (lowering versus lifting) and grasp phases (IFO versus MFA versus FFO). In all analyses post-hoc comparisons were performed using the Newman-Keuls procedure. The significance level was fixed at p=0.05.

### Results and Discussion

The mean values of the parameters collected in experiment 4 are reported in Table 1. RT (F(2,26)=7.8, p=0.002), peak acceleration (F(2,26)=7.4, p=0.003) and peak velocity (F(2,26)=9.28, p=0.001) of both foot-tip lowering and lifting were affected by factor grasp phases. Figure 2 and post hoc comparisons show that IFO phase induced a significant increase in RT with respect to the other two phases (MFA p=0.003, FFC p=0.005) which did not differ from each other (p=0.88); peak acceleration was significantly smaller in IFO phase than MFA and FFC phases (p=0.04, p=0.002). Peak velocity was significantly smaller in IFO phase than in both the MFA (p=0.008) and FFC phase (p=0.0008). For both parameters the latter two phases did not differ from each other (p=0.1, p=0.18). 

Factor foot movement showed significance only for peak velocity (F(1,13)=14.19, p=0.002, 574 mm/s, lowering, 495 mm/s lifting).

The results of experiment 4 demonstrated that the effects of the observation of the various grasp phases affected movements of even the foot. The final grasp phases made quicker foot movements regardless of type of movement. 

In previous studies [12–14] we found that type of grasp observation modulated voice spectra parameters of syllables pronounced during observation. However, it was not investigated the influence of grasp phases on speech. 

## Experiment 5

We were interested in determining which grasp phase was responsible for maximal effect on syllable pronunciation.

### Methods

#### Participants

A new sample of fourteen volunteers (14 females, 20-30 years) participated in experiment 5 after providing written informed consent. All were right-handed, as ascertained by the Edinburgh Handedness Inventory [18]. All participants were naïve as to the purpose of the study. The Ethics Committee of the Medical Faculty at the University of Parma approved the experiment, which was carried out according to the declaration of Helsinki.

#### Apparatus stimuli and procedure

The participants seated in front of a table with their hands resting upon the table plane. Each trial began presenting a sound (BEEP; duration 500 ms). Then, on a PC monitor placed on the table plane (19 inches) 60 cm distant from the participant’s chest, the participant was presented with a syllable printed on the PC display centre (Figure 1 /ba/ or /da/; character arial, 60 pts, bold) lasting 500 ms, followed by one picture of the following grasp postures: IFO, MFA, FFC of grasping either the apple or the peach (Figure 1). Each picture whose duration of presentation was 2000 ms was preceded by another picture in which the hand was presented in the initial pinch position (200 ms duration). The participant was required to pronounce the presented syllable once the hand was perceived as still. After picture presentation a blank panel was presented for 4000 ms before the successive trial.

#### Movement recording, voice recording and data analysis

The lip movements were recorded by the SMART system (BTS Bioengineering, Milano, Italy). In order to study the lip kinematics we attached one marker (5 mm diameter) to the upper lip, and another to the lower lip. We measured for successive statistical analysis, RT, that is the time from the presentation of the picture to the beginning of lip opening, and in addition peak acceleration and peak velocity of lip opening.

The participants wore a light-weight dynamic headset microphone (Shure, model WH20). The frequency response of the microphone was 50–15,000 Hz. The microphone was connected to a PC by a sound card (16 PCI Sound Blaster; CREATIVE Technology Ltd., Singapore). We acquired voice data of the participants during syllable pronunciation using the Avisoft SAS Lab professional software (Avisoft Bioacoustics, Germany), and calculated the participants’ voice parameters using the PRAAT software (www.praat.org). In particular, we analyzed the time course of formant (F) 1 and 2 of the syllable vowel. It is well known that F1 and F2 define vowels from an acoustical point of view exactly (Leoni and Maturi, 2002). Both formant transition and pure vowel pronunciation were measured and averaged. 

Repeated measures ANOVAs were carried out on F1, F2, RT, lip peak acceleration, and lip peak velocity. The within-subjects factors were syllable (/ba/ versus /da/) and grasp phases (IFO versus MFA versus FFC). In all analyses post-hoc comparisons were performed using the Newman-Keuls procedure. The significance level was fixed at p=0.05.

### Results and Discussion

**Figure 3:**
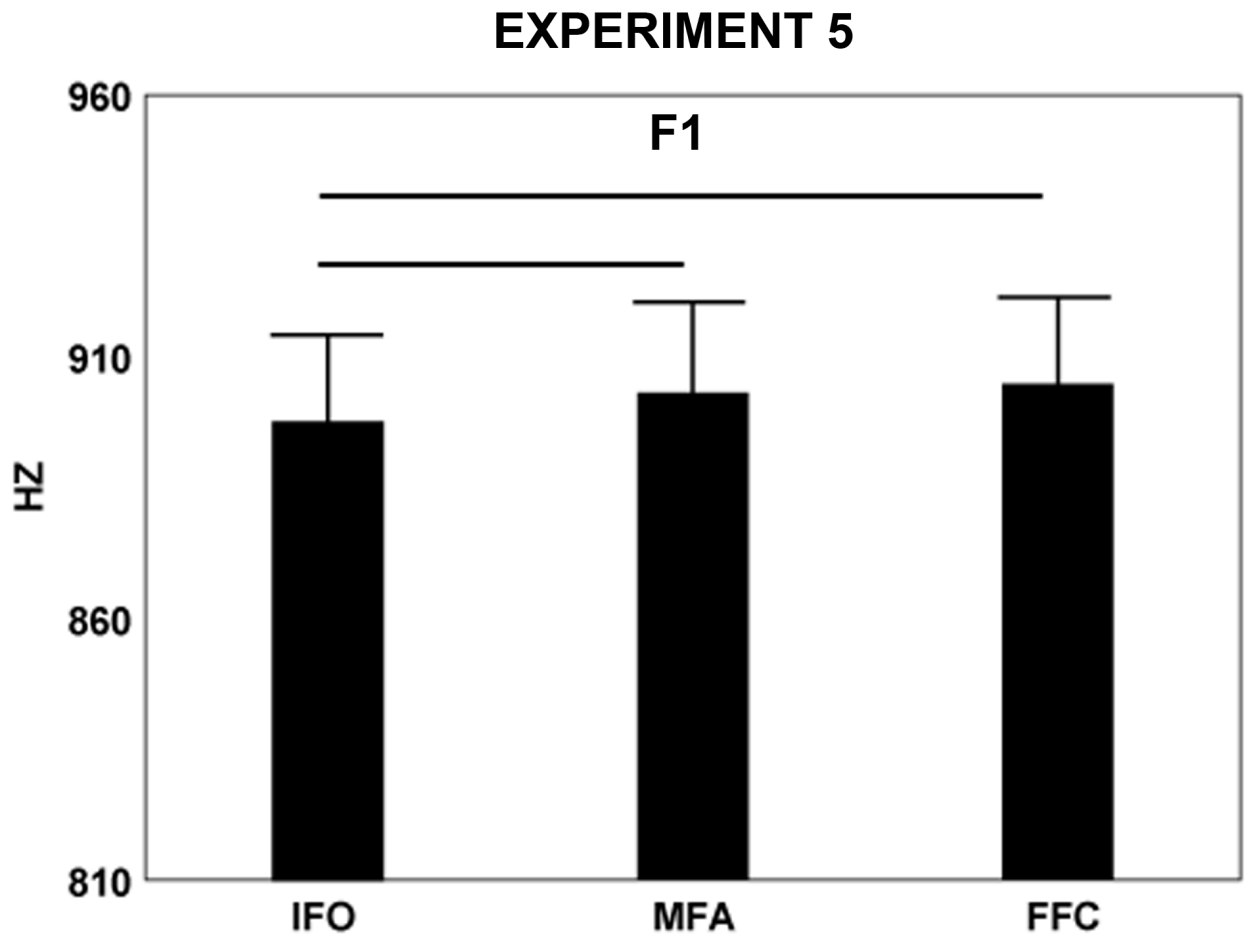
Mean RT and voice spectra parameter (F1) of the syllable /ba/ and /da/ after presentation of three phases of grasp (IFO, initial finger opening, MFA, maximal finger aperture, FFC, final finger closing, experiment 5). Other conventions as in Figure 2.

Table 1 reports the mean values of F1 collected in experiment 5. F1 was affected by factor grasp phases (F(2,26)=5.1, p=0.014). Figure 3 and post-hoc comparisons show that this parameter significantly decreased in IFO phase than in both MFA (p=0.03) and FFC phases (p=0.01). The latter two phases did not differ from each other (p=0.46). Factor syllable affected F1 and F2 (F1, F(1,13)=57.1, p=0.0001, /ba/ 910 Hz, /da/ 893 Hz; F2, F(1,13)=116.80, p=0.00001, /ba/ 1434 Hz, /da/ 1550 Hz). Peak acceleration (F(1,13)=30.0, p=0.0001, /ba/ 4833 mm/s^2^, /da/ 2986 mm/s^2^) and velocity of lip opening (F(1,13)=75.4, p=0.00001, /ba/ 207 mm/s, /da/ 115 mm/s) were higher when pronouncing /ba/ as compared to /da/. RT was affected by factor syllable (F(1,13)=6.30, p=0.026, /ba/, 379 ms, /da/, 305 ms) .

Results confirmed an effect of grasp observation on F1 [13,14]. This was maximal during the observation of the final closing phase. As previously found [e.g. 18] F1 increased and F2 decreased when pronouncing /ba/ as compared to /da/.

The grasp phases did not affect RT, peak acceleration and velocity of lip opening. We expected an effect especially on /ba/ when the lips actively participate in syllable pronunciation [11–13]. However, the control of the external mouth might be less sensitive to grasp phases than internal mouth whose contribution to syllable pronunciation is stronger [19].

The previous experiments left unsolved the following issues; the first is if the observer simulated the grasp. In affirmative case, the second issue was if we observed either interference during simulation of the initial phase of grasp which was removed during the final grasp phase or facilitation during simulation of the grasp final phase. The third issue related to the first one was if the stronger command to execute the actual movement (whose result was an increase in the kinematic parameters) corresponds to stronger activation of motor area involved in simulation of the observed movement phase. To solve these issues we used TMS technique and stimulated hand M1 when participants observed the various phases of grasp. We expected variation in MEPs during observation of grasp phases as compared to a control condition in which the fruit was presented alone (first issue). We expected either a decrease in MEPs during the initial grasp phase or an increase during the final grasp phase (second issue). In addition (third issue), an increase/decrease in MEPs should correspond to an increase/decrease in kinematic parameters of the actual movement found in the behavioural experiments of the present study.

## Experiment 6

We determined whether the effect of observation of grasp phases induced activation of observer’s hand M1 (i.e. simulation of hand movement).

### Methods

#### Participants

A new sample of ten right-handed [18], naïve and Italian native speakers (8 females, age 20-30 yrs.) took part in the experiment. All participants were screened to rule out any history of neurological, psychiatric, or medical problems, and to check for possible contraindications to TMS [20]; they signed consent form and were paid for their participation. The Ethics Committee of the Medical Faculty at the University of Parma approved the experiment, which was carried out according to the declaration of Helsinki. 

#### TMS

The experiment took place in a soundproofed room where participants sat in front of a PC monitor (19 inches) placed on the table 100 cm distant from the participant’s chest. The participants were seated on a comfortable armchair, with their elbow flexed at 90° and their hands prone in a relaxed position. To avoid any movement that would have influenced the positioning of the stimulation, they leaned their chin on a chin-support. Single pulses TMS were delivered using one module of a Bistim system (Magstim Co. Ltd.) and using a 70 mm figure-of-eight standard coil (Magstim Co. Ltd.). The coil was held tangential to the head. A mechanical coil holder allowed the placement of the TMS coil in the desired position. TMS single pulses were applied to the hand area of the left M1 in order to evoke a response in the contralateral OP, chosen because of its involvement in grasping movement [14]. An EMG was recorded in order to quantify MEP amplitudes. First, the individual resting motor threshold (RMT) was assessed. The RMT was defined as the stimulation strength (in percentage out of machine output) at the "hot spot" which led to EMG amplitudes above 100 μV in 50% of 10 pulses [21]. The optimal location for eliciting MEPs in the contralateral OP was marked on the scalp and the TMS coil was fixed exactly above this point. Participants wore a swimming cap on the head to allow for accuracy in marking the site for stimulation. For the experiment, a stimulation intensity of 120% above the individual motor-threshold was used. Subjects were asked to relax muscles completely during TMS application and task performance. Furthermore, as a criterion of trial acceptance, we checked for the absence of any detectable EMG activity for the entire duration of each trial (except after stimulation). 

#### Apparatus, stimuli and procedure

**Figure 4:**
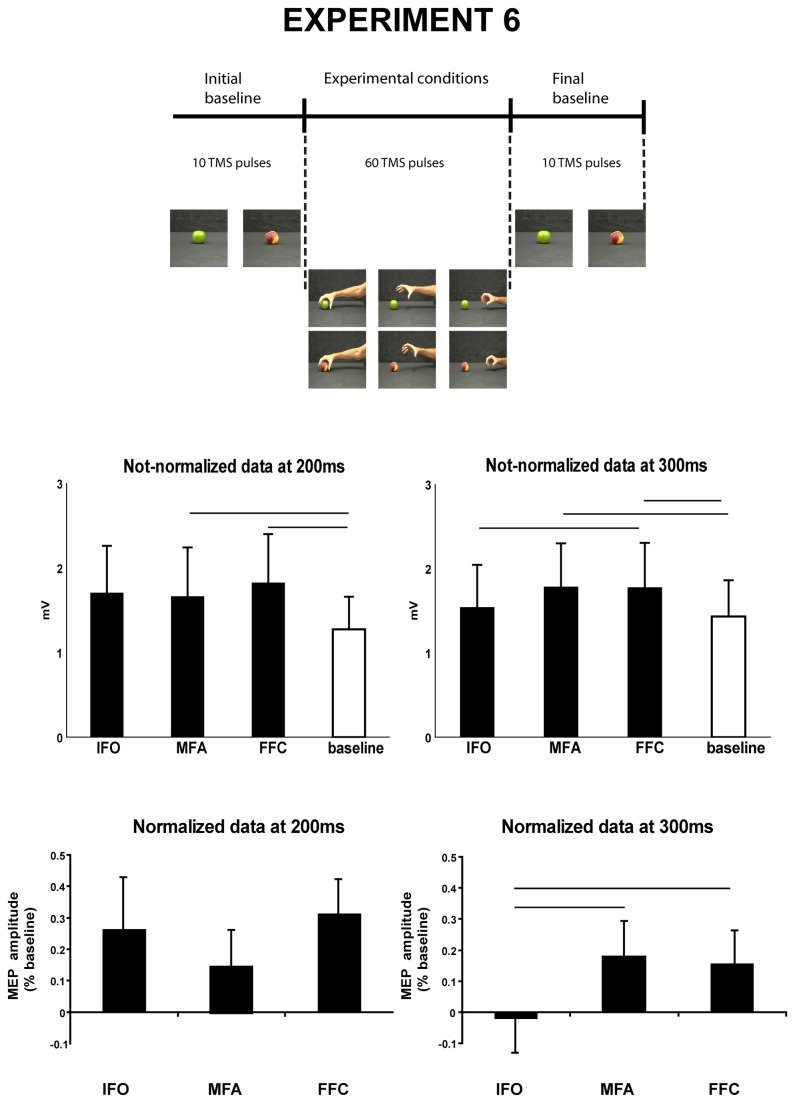
MEPs recorded after TMS applied to M1 during observation of three phases of grasp (IFO, initial finger opening, MFA, maximal finger aperture, FFC, final finger closing, experiment 6). The upper panel shows the stimuli. TMS was applied 200 and 300 ms post-stimulus. Not normalized data and data normalized with respect to a baseline condition are presented. Other conventions as in Figure 2.

Each trial started with a BEEP and then the participant was presented with one picture of the following grasp postures: IFO, MFA, FFC (Whole Hand Grasp of either an apple or a peach, Figures 1 and 4). Each picture whose duration of presentation was 1500 ms was preceded by another picture in which the hand was presented in the initial pinch position (200 ms duration). TMS was randomly applied at 200 and 300 ms after presentation of each phase in order to identify when the variations in M1 activity occurred during stimuli processing. Then a blank panel was presented for 5000 ms (Figure 1). Only when a question mark appeared on the screen 500 ms after stimuli offset (10% of trials, randomly distributed), they were required to state aloud the stimuli observed in the last trial. The participants were instructed to pronounce “initial hand” (*mano iniziale*, IFO) -“opened hand” (*mano aperta*, MFA) -or-“closed hand” (*mano chiusa*, FFC) in response to the grasp phases. 60 trials were presented, 10 trials for each condition (3 grasp phase pictures and 2 TMS delays). A baseline measurement preceded (initial baseline, 10 trials) and followed (final baseline, 10 trials) the experimental conditions (see Figure 1, Figure 4). For this, participants were required to fixate the fruit (an apple or a peach) on the screen while TMS stimulation was applied at 200 or 300 ms post-stimulus. In initial and final baseline five TMS at 200 ms post-fruit-stimulus and five TMS at 300 ms post-fruit-stimulus were randomly applied. Baselines1 and 2 refer to MEPs recorded after TMS at 200 and 300 ms post-fruit-stimulus. In total, 80 trials were run. 

#### Data recording and analysis

Continuous EMG recordings from OP was acquired with a CED Micro 1401 (Cambridge Electronic Design, Cambridge, U.K.) connected to CED 1902 amplifier and interfaced with CED Spike software. EMG signal was amplified (1000×), digitized (sampling rate: 2.5 kHz) and band-pass filtered (5–5000 Hz). The muscle was recorded with a pair of disposable Ag–AgCl surface electrodes (diameter 3mm). The active and reference electrodes to record EMG signal were placed on the belly and the distal tendon of the muscle, respectively. The ground electrode was placed over the participants' left wrist and connected to the common input of the CED input box. (CED 1902, CED Ltd.). Visualisation and later processing was done using Spike2 software (CED Ltd.). 

The peak-to-peak amplitude (mV) of each MEP was computed by using Matlab software. MEP amplitudes less than 50 μV were not considered. Normalized MEPs were calculated as variation of each value of the first (200 ms) and the second (300 ms) TMS delay with respect to the mean of the baseline1 and 2 values respectively (MEPnorm=(MEPrecorded- MEPbaseline) /MEPbaseline). MEP means of not-normalized and normalized data in each condition were submitted to repeated measures ANOVAs with grasp phases (IFO versus MFA versus FFC, baseline 1 or 2 for data not normalized) and stimulation-delay (200 versus 300 ms) as within-subjects factors. All post hoc comparisons were carried out using Newman-Keuls test. Significance was established in all analyses at p=0.05. 

### Results and Discussion

All the vocal responses were correct.

#### Analysis of the not-normalized data

The ANOVA on mean MEP peak-to-peak amplitudes revealed a significant effect of factor grasp phases at both 200 and 300 ms post-stimulus TMS delivery (F(3,27)=3.38, p=0.032; F(3,27)= 4.4, p=0.012, Figure 4). 

At 200 ms post stimulus TMS, post-hoc analyses showed that the grasp phases processing elicited in all the conditions (IFO = 1.7 mV, MFA = 1.7 mV, FFC = 1.8 mV) greater MEP amplitude compared with the baseline1 (1.3 mV, p = 0.044, p= 0.027, note that a trend to significance was found for the comparison between baseline1 and IFO phase p=0.065), whereas MEP amplitudes among the three phases did not differ from each other (p>=0.52). At 300 ms post-stimulus TMS, post-hoc comparisons revealed a higher MEP for MFA (1.8 mV) and FFC (1.8 mV) when compared with baseline2 (1.4 mV, p=0.029, p=0.019). No difference was observed in MEP amplitude when the baseline2 was compared with IFO condition (p=0.38, 1.5 mV). Furthermore, the MEP amplitude in FFC was significantly higher than in IFO (p=0.05). 

#### Analysis of the normalized data

No significant changes were reported in the ANOVA at 200ms post stimulus TMS delivery (F(2,18)=8.14, p=0.46). At 300 ms post stimulus TMS delivery, a significant effect of grasp phases was, on the contrary, observed (F(2,18)=6.5, p=0.008, Figure 4). Post hoc comparisons revealed that MEP amplitudes in IFO were lower than in the other two grasp phases (p=0.01; p=0.01) which did not differ from each other (p=0.68).

The results of the present experiment show an initial unspecific TMS effect (200 ms post stimulus) concerning the grasp phases; the observation of the grasp activated contraction of OP muscle which was not yet modulated through the grasp phases. In contrast, the stimulation at 300 ms induced greater activation during maximal finger aperture and final finger closure as compared to initial finger opening. 

## General Discussion

### Behavioural experiments

We assumed that the observation of a specific grasp phase is better coupled with the beginning of a successive movement of the observer when, in the comparison with other phases, RT decreases and/or kinematic parameters of the successive movement (e.g. acceleration and velocity) increase. The results of experiments 1-4 showed that the observation of three phases of grasping a fruit selectively affected the kinematics of successive finger and foot movements. The observed MFA and FFC phases induced a decrease in RTs, and an increase in acceleration and velocity of the actual finger opening/closing as compared to observed IFO. The finding that the start of actual finger (and foot) movement was coupled with observation of final finger closing (FFC) rather than initial finger opening (IFO) indicates that concatenation rather than synchronism relates observed grasp to executed movement of distal effectors. In fact, a synchronism should facilitate finger opening after observation of IFO, and finger closing after observation of MFA. Conversely, concatenation should facilitate the successive movement after observation of FFC, that is the final grasp phase. On a social point of view, concatenation may be chosen in order to act without interfering with the conspecific’s behaviour. In contrast, synchronism is more related to resonance behaviours, and probably resonating movements between individuals reflect a more primitive stage of social interactions. 

Experiment 2 ruled out the possibility that this effect was due to salience of the visual stimulus when the hand was close to the target during the final finger phases. Moreover, it suggested that the pantomime of grasp postures influenced the successive finger opening as well as the grasp did. This may disprove the possibility that in experiment 1 the achieved aim of the observed grasp triggered the successive action. However, the presence of a fruit and a postured hand in the scene, even if in incongruent locations for a grasp, might also evoke an aim (i.e. taking possession of the object). In general, the data suggest that the observation of the final phases of grasp of a conspecific made quicker the observer’s movement. We propose that the observation of a grasp posture induced simulation of the movement in order to assume that posture (see results of experiment 6). The simulation of successive phases of grasp gradually facilitated the execution of successive movements and it was maximal when the action was to be accomplished. This induced variation in RTs and kinematic parameters (velocity and acceleration). Facilitation could occur in advance in comparison with grasp end (i.e. during MFA). This is in agreement with the idea proposing preparation of a successive movement in motor chains when the previous one was not yet accomplished [4]. In other words, actual finger opening (or closing) could be activated when the observed fingers started to close. The data of the TMS experiment 6 confirm that the observation of successive grasp phases induced an increase (facilitation) in MEPs. Facilitation made actual observer’s finger opening faster as shown by the behavioural results.

The presentation of grasp postures without any apparent motion induced effects similar to those when the apparent motion was added. However, the effect was weaker and less specific, because grasp phases modulated RT and peak acceleration rather than peak velocity and it was present for both finger opening and closing. We interpret these results as consequent mainly to inconstant simulation of the observing posture. This interpretation may be also due to instructions provided to the participants. In experiment 1 they were required to start to move when they judged that the presented hand was still, whereas in experiment 3 when a still hand was presented. In other words, it was stressed analysis of stimuli motion in experiment 1, and of static stimuli in experiment 3. This might induce motor simulation in experiment 1 rather than in experiment 3. Nevertheless, simulation was present even for still posture even if less evident than for apparent motion. This is in agreement with previous data showing automatic imitation of the kinematics of distal effectors [7,9] and effects of postures of distal effectors on movements executed with another distal effectors [22]. 

The effect of grasp observation was found during actual finger opening/closing and during movements of foot-tip lowering/lifting. However, in experiment 2 the effect was selective for finger opening. The lack of an effect on actual finger closing was probably due to the fact that the simulated final position of the fingers (closed) was incompatible with commands of finger closing because the fingers were already closed. The fact that it was found especially in experiment 2 may depend on presentation of finger opening/closing without any target object in a grasp-compatible position. These movements were more similar to those successively executed. Thus, the start signal could be functionally related to simulation when the same effectors and the same type of movement were involved in both observation and actual movement (finger opening command could be concatenated to closed rather than open simulated posture). In contrast, when different effectors and types of movement were involved, the command was unspecific.

Observing the grasp phases affected pronunciation of even syllables. F1 was higher when the final phase of grasp was observed. This result is in agreement with previous data which demonstrated that the observation of different grasps of objects affected vocal parameters of syllables pronounced during observation [12–14]. The results of the present study, in addition, suggest that the command to the mouth was stronger when the observed grasp was in the final finger closing phase. In experiment 1, concatenation was present when controlling simple movements (opening and closing) as well as complex movements (speech). Consequently, concatenation may be also specific when the two movements are related to each other. For an example, finger opening is related to finger closing rather than grasp. Conversely, the control of phonatory organs when pronouncing syllables is related to grasp [16] but when pronouncing vowels is related to finger opening [23]. 

### TMS experiment

TMS experiment showed an increase in MEPs of OP muscle during grasp observation which reached the maximal value during the final phases. The present data are in accordance with the results of the study by Gangitano et al. [17]: these authors found a gradual increase in FDI during observation of the initial grasp phase which reached a maximum during maximal finger aperture. 

TMS selectively affected FDI [18] and OP according to grasp phases: MEPs of FDI increased during the finger opening phase, whereas MEPs of OP increased during the finger closing phase. In contrast, in the behavioural experiments the observation of the final phase of grasp affected contraction of agonistic/antagonistic muscles of the same or different distal effector. We can explain these results as due to simulation of finger aperture/closure that was used to concatenate the grasp to the successive movement. This process was automatic and unaware because the participants were not instructed to simulate and to start to move after completion of grasp simulation. Nevertheless, they might automatically estimate the time to contact simulating the grasp from the initial hand posture to the presented hand posture. If the time was brief, RT decreased and acceleration and velocity of the successive actual movement increased 

## Conclusions

The mechanisms joining the observation of conspecific’s action with observer’s actions may be precursor of social functions. They may be at the basis for interaction behaviours between conspecifics. This hypothesis is suggested by the finding that the observation of the final phase of an action executed by an individual maximally activated the muscles involved in the successive action of another individual. Indeed, this mechanism couples in sequence the execution of two actions executed by two different individuals.

The second possible function of this mechanism is related to communication. The data of the present study indicate that the observation of an action influences the vocal parameters of syllables pronounced during action execution. This is in agreement with previous studies in which the observation of reach to grasp [12–14] and bring to mouth [24] affected vocal spectra of syllables pronounced during observation. Similarly, the observation of symbolic and representational gestures [25–27] affects the vocal spectra of word and syllables pronounced during gesture production. It is possible that gesture aspects are embedded in voice features [16,28]. However, it is also possible that gesture observation automatically activates an observer’s verbal response which was concatenated to gesture (experiment 5).
